# A Single-Day Treatment with Mifepristone Is Sufficient to Normalize Chronic Glucocorticoid Induced Suppression of Hippocampal Cell Proliferation

**DOI:** 10.1371/journal.pone.0046224

**Published:** 2012-09-25

**Authors:** Pu Hu, Charlotte Oomen, Anne-Marie van Dam, Jordi Wester, Jiang-Ning Zhou, Marian Joëls, Paul J. Lucassen

**Affiliations:** 1 Swammerdam Institute for Life Sciences, Center for Neuroscience, University of Amsterdam, Amsterdam, The Netherlands; 2 CAS Key Laboratory of Brain Function and Diseases, School of Life Science, University of Science and Technology of China, Hefei, Anhui, China; 3 VU University Medical Center, Neuroscience Campus Amsterdam, Department of Anatomy and Neurosciences, Amsterdam, The Netherlands; 4 Rudolf Magnus Institute for Neurosciences, Department of Neuroscience and Pharmacology, University Medical Center Utrecht, Utrecht, The Netherlands; Radboud University, The Netherlands

## Abstract

**Background:**

Chronic stress or prolonged administration of glucocorticoids suppresses proliferation and/or survival of newborn cells in adult rat dentate gyrus. Earlier we showed that administration of the glucocorticoid receptor antagonist mifepristone during the final 4 days of a 21 days period of corticosterone treatment fully normalized the number of newborn cells. Here we aimed to better understand how mifepristone achieves this effect and questioned whether an even shorter (single day) mifepristone treatment (instead of 4 days) also suffices to normalize neurogenesis.

**Methods:**

We investigated various steps of the neurogenic process, using the immunohistochemical markers BrdU, doublecortin, proliferating cell nuclear antigen as well as glial fibrillary acidic protein, after 17 or 21 days of corticosterone (versus vehicle) treatment.

**Results:**

Corticosterone primarily attenuates the proliferation of cells which subsequently develop into neurons; this is fully reversed by mifepristone. Surprisingly, the corticosteroid effects on neurogenesis can even be fully re-set by a single-day treatment with mifepristone (on day 18), despite the continued corticosterone exposure on subsequent days.

**Conclusions:**

Our results emphasize that studies into the therapeutical efficacy of new antidepressants, especially those targeting HPA-activity or the glucocorticoid receptor, should explore the possibility to reduce treatment duration.

## Introduction

Exposure to stress leads to activation of hypothalamo-pituitary-adrenal axis (HPA), eventually resulting in enhanced release of glucocorticoid hormones from the adrenal. These hormones enter the brain and bind to intracellular receptors [1]. Glucocorticoid receptors (GRs) are enriched in limbic areas like the hippocampus and, due to relatively low affinity for corticosterone, are primarily activated after stress [2].

Chronic stress and HPA dysfunction are generally considered risk factors for the development of psychiatric disorders, including major depression [3], [4], [5], [6]. For instance, HPA-axis hyperactivity is often seen in depressed patients and even in healthy high-risk proband with a positive family history for affective disorders [7], [8], [9]. HPA dysfunction is partly normalized upon treatment and the degree of normalization inversely correlates with relapse probability [10]. Recently, individuals with severe types of depression, e.g. psychotic depression, were reported to benefit from treatment with the GR-antagonist mifepristone [11], [12], [13], [14].

The cellular effects of chronic stress in the brain have been extensively studied in rodent models, for reviews see [15], [16], [17], [18]. Many parameters in target areas of corticosteroids, e.g. the hippocampus, are altered after 21 days of stress or treatment with high doses of corticosterone (the prevailing rat glucocorticoid), including neurogenesis in the dentate gyrus (DG), for reviews see [16], [19], [20], [21], [22]. Adult neurogenesis refers to the process by which stem cells located in the subgranular zone undergo sequential stages of proliferation, migration and neuronal differentiation before incorporated into the existing adult hippocampal network [23], [24], [25], [26]. Chronic stress and corticosterone treatment were reported to reduce cell proliferation [27], [28], [29], [30], neuronal differentiation [31] and/or survival of newborn cells [32] although also exceptions have been reported, for reviews see [20], [33], [34]. Interestingly, the stress-reduced neurogenesis could be completely normalized by mifepristone administration during final 4 days of stress or corticosterone administration (i.e. on days 18–21), whereas the drug was ineffective in the handled control group [35], [36]. This may bear relevance to the clinical efficacy of mifepristone.

*How* mifepristone achieves this normalizing effect is not well understood. We performed two experiments to obtain more insight. If corticosterone would only increase vulnerability to cell death until day 18 while the actual reduction in newborn cell number would only take place between days 18–21, then mifepristone treatment starting at day 18 might prevent the latter from happening (a ‘rescue’ effect). On the other hand, if corticosterone would systematically reduce survival of newborn cells throughout the entire application period, the normalizing effect of mifepristone might take place between days 18–21, e.g. by promoting additional rounds of cell division. In the first scenario, the number of surviving newborn cells up to 17 days of corticosterone administration is expected to be comparable to that in vehicle-treated controls. In the latter case the number of surviving newborn cells will be reduced after 17 days of corticosterone. Corticosterone might also preferentially attenuate *proliferation*, which would then be prevented or reversed by mifepristone. These possibilities were examined in the first experiment, by systematically studying cell proliferation and survival after 17 or 21 days of corticosterone/vehicle administration. In the second experiment we questioned whether mifepristone treatment for 4 consecutive days is necessary, or whether a single-day treatment is already sufficient to reverse the chronic corticosterone effect.

## Materials and Methods

### Animals

All animal procedures presented in this paper were approved by the animal ethics committee of the University of Amsterdam. We here report on data obtained in 48 adult male Wistar rats (8 weeks of age; 180–200 g on arrival). All animals were housed in pairs under controlled conditions of a 12/12 h light/dark cycle (lights on 08:00 h) with food and water *ad libitum*. They were habituated to the experimental setting for 10 days. Temperature and humidity were kept at 20–22°C and 50–55% respectively.

### Corticosterone and mifepristone treatment

Corticosterone (CORT; Sigma, C-2505; 40 mg/kg) was dissolved in arachidus oil. CORT or vehicle (VEHC) was subcutaneously injected daily at 09:00 h for 17 days or 21 days. Mifepristone (50 mg/kg body weight; Sigma, St Louis, MO, USA) was dissolved in 15 µL ethanol/1.5 mL coffee cream (Campina, Woerden, The Netherlands). In experimental groups examined for 21 days, animals were treated either i) only on day 18 or ii) on days 18–21, both at 09:00 h and 16:00 h with mifepristone or its vehicle (VEHM), administered through an oral syringe directly into stomach.

**Figure 1:**
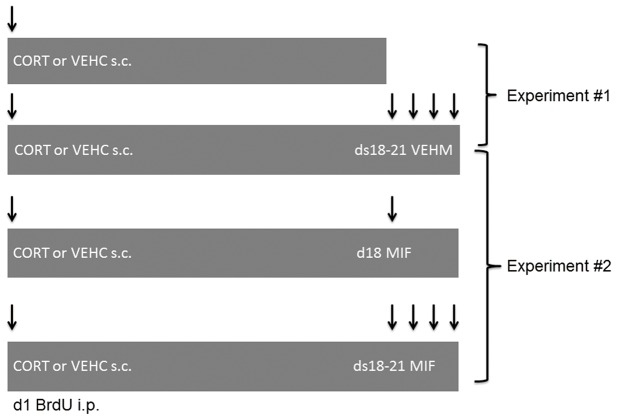
Schematic representation of experimental groups. In experiment #1, we compared animals treated subcutaneously with corticosterone (CORT) or vehicle (VEHC) daily for 17 days with animals treated for 21 days; the latter groups also received the vehicle of mifepristone (VEHM) on days 18–21. In experiment #2, the 21 ds CORT/VEHC+ds 18–21 VEHM were used as statistical reference groups in a comparison with experimental groups receiving mifepristone on d18 only (21ds CORT+ds18 MIF and 21ds VEHC+ds18 MIF) or on 4 days from 18–21 (21ds CORT+ds18–21 MIF and 21ds VEHC+ds18–21 MIF). All animals were treated with BrdU on day 1 and sacrificed on the morning after the last treatment.

Rats were randomly assigned to one of eight experimental groups (n = 6 animals per group; see Figure 1 for schedule). Comparable to our earlier study [36], one group received corticosterone injections for 21 days and another group received 21 days of corresponding vehicle. Both groups were treated with the vehicle of mifepristone on days 18–21 (21ds CORT+ds18–21 VEHM and 21ds VEHC+ds18–21 VEHM respectively). In view of the high reproducibility of corticosterone-induced reduction in neurogenesis [31], [32], [36], it was considered valid to use these two groups as a statistical reference for the two experiments, which is also in accordance with the European animal research ethics law that aims for reduction in the number of experimental animals as much as possible.

For experiment #1, the two reference groups were compared with a 17 days CORT group (17ds CORT) and a 17 days VEHC group (17ds VEHC). For experiment #2, we added four groups: rats received mifepristone treatment during the final 4 days of corticosterone or vehicle (21ds CORT+ds18–21 MIF; 21ds VEHC+ds18–21 MIF respectively); and rats receiving 21 days corticosterone or vehicle, in combination with mifepristone treatment on day 18 only (21ds CORT+d18 MIF and 21ds VEHC+d18 MIF, respectively). During treatment all experimental groups were mixed and animals were sacrificed on next day after the last treatment (i.e. on d18 or d22 for the 17ds and 21 ds treatment groups, respectively).

### Body weight

Body weights were measured regularly, first at the beginning of experiment to assess the baseline, and subsequently at daily intervals. Data are expressed in percentage change of body weight (weight at the day of sacrifice minus the baseline value divided by baseline value).

### Bromodeoxyuridine labeling

To study survival of newborn cells, all animals received 5-bromo-2-deoxyuridine (BrdU) intraperitoneally (200 mg/kg body weight, dissolved in 0.9% saline) at noon on the first day of chronic CORT or vehicle administration (i.e., 3 h following the first CORT or VEHC injection). The single injection paradigm was chosen to ensure that the delay between, and the acute effect of, the first injection (of CORT or VEHC) and BrdU incorporation was comparable for all animals.

### Brain tissue processing

At the day of sacrifice, animals were anaesthesized in the morning with pentobarbital sodium salt (Nembutal, 1 mg/kg bodyweight; A.U.V. Cuijk, the Netherlands) and perfused transcardially with saline followed by 4% paraformaldehyde in 0.1 M phosphate buffer (PB, pH 7.4). To prevent pressure artefacts, brains were additionally post-fixed overnight in the skull at 4°C, washed and cryoprotected in 30% sucrose in PB. Frozen sections (40 µm thick) were cut using a sliding microtome and collected in PB with sodium azide.

### Immunohistochemistry

Different stages of neurogenic process were studied as described previously [37]. Immunohistochemistry for BrdU (monoclonal mouse anti-BrdU, Roche Diagnostics, the Netherlands; 1∶1000) was used to assess cell survival of proliferating cells marked at the first day of corticosterone (or vehicle) administration; PCNA (monoclonal mouse anti-PCNA, DAKO, 1∶400) to assess proliferation; and doublecortin (DCX) (polyclonal goat anti-DCX, SantaCruz; 1∶800) to estimate neurogenesis. To analyze changes in astrocyte numbers in dentate gyrus, immunohistochemistry for GFAP (polyclonal rabbit anti-GFAP, DAKO; 1∶10000) was done as well. Amplification was performed with biotinylated secondary antibodies, sheep anti-mouse (1∶200; GE Healthcare), donkey anti-goat (1∶500; Jackson ImmunoResearch Labrotories) and goat anti- rabbit (1∶200; Vector Laboratories) immunoglobulins respectively, followed by incubation in avidin-biotin complex (1∶1000; Elite Vectastain ABC kit, Brunschwig Chemie, Amsterdam) and biotinylated tyramide (1∶500; 0.01% H_2_O_2_; kindly provided by Dr. I. Huitinga, Netherlands Institute for Neuroscience, Amsterdam) and avidin-biotin-complex. Chromogen development was performed with diaminobenzidine (DAB; 20 mg/100 mL Tris buffer, pH 7.55, 0.01% H_2_O_2_).

To assess whether DCX^+^ neurons can re-engage in cell cycle and undergo again proliferation, additional double immunofluorescence stainings were performed for Ki67 and DCX in a limited set of tissue sections from 21ds CORT+d18 MIF rats and their vehicle treated controls. The following antibodies and conditions were used; DCX (SantaCruz, polyclonal made in goat; 1∶150) and Ki67 (Novocastra, polyclonal made in rabbit; 1∶250). Mounted sections were first heated in 0.01 M citriate buffer (pH 6.0) in a microwave oven (Bosch) for 5 minutes at 800 W and then 5 minutes at 400 W followed by 5 minutes at 260 W. After a cool down period outside the oven of approximately 20 minutes, the sections were washed and incubated in primary antibodies diluted in 0.25% gelatine and 0.5% Triton-100 at room temperature for 1 h, and then incubated overnight at 4°C. The next day, the sections were washed and incubated for 2 h with donkey-anti-rabbit biotinylated secondary antibody (for Ki67, Jackson ImmunoResearch Labrotories; 1∶200) and donkey-anti-goat Alexa 488 antibody (for DCX, Molecular Probes, Leiden, The Netherlands; 1∶200) at room temperature before storage overnight at 4°C. The next day sections were washed and incubated for 2 h in Alexa 488-labeled streptavidine antibody (for Ki67, 1∶400, Molecular Probes, Leiden, The Netherlands; 1∶400, green). Following a brief rinse, they were embedded in Vectashield (Vector Laboratories). Fluorescent signal was detected using a confocal Nikon A1 laser scanning microscope and simultaneously collected.

### Quantification of DCX, GFAP, BrdU and PCNA

Stereological quantification of the number of DCX^+^ cells and GFAP^+^ cells was performed unilaterally by systematic random sampling in every 10^th^ section using the StereoInvestigator system (Microbright field, Germany) according to stereological principles described previously, for details see [38] and without a left/right preference within or between animals. Because of relatively low numbers and the occurrence of clusters, BrdU^+^ and PCNA^+^ cells were counted manually by means of modified stereological procedure in every 10^th^ hippocampal section (Zeiss microscope 200× magnification) and multiplied by 10 to estimate the total number in DG.

### Statistics

Data are presented as mean ± SEM. All statistics were performed with SPSS 16. Data were subjected to an ANOVA, using p<0.025 as the level of significance, thereby correcting for the double use of the references groups (21 ds CORT and 21 ds VEHC). This was followed by a post-hoc Tukey multiple comparisons of the means.

## Results

### General expression patterns of BrdU, DCX, PCNA and GFAP

**Figure 2:**
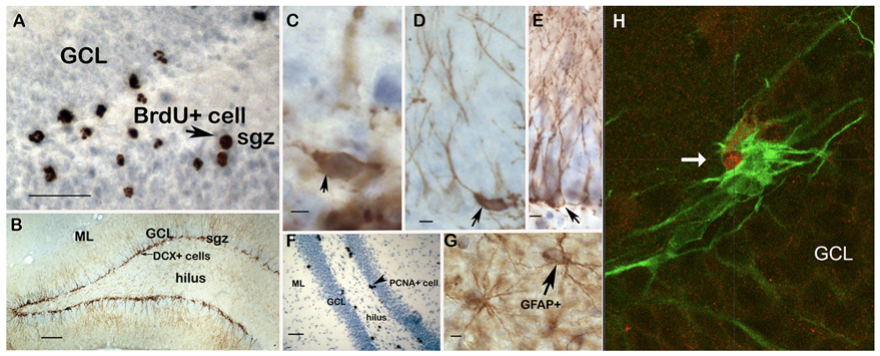
Distribution of BrdU, DCX, PCNA and GFAP positive cells in the rat dentate gyrus. **A.** BrdU^+^ cells are mainly located in the subgranular zone (SGZ, arrowhead) but can also be found in the hilus. Considerably lower numbers are encountered in the granular cell layer (GCL). Calibration bar: 50 µm. **B.** Overview of the rat dentate gyrus with vehicle treatment showing strong immunoreactivity of DCX in the SGZ and GCL, with dendrites extending through the GCL into the molecular layer (ML). Calibration bar: 100 µm. **C.** Arrowhead points to a relatively immature type 1 DCX^+^ cell, without dendrites and/or with horizontally oriented dendrites. Calibration bar: 10 µm. **D.** Arrowhead points to a type 2 DCX^+^ cell, with dendrites with an oblique orientation, growing into the GCL but not ML. Calibration bar: 10 µm. **E.** Arrow points to a relatively mature type 3 DCX^+^ cell, characterized by a primary dendrite orientated perpendicularly to the SGZ and with protrusions extending into the ML. Calibration bar: 10 µm. **F.** Clustered PCNA-labeled cells prevail in the SGZ (arrowhead) but can also be found in the hilar region; these cells are less prevalent within the GCL. Calibration bar: 50 µm. **G.** GFAP^+^ astrocytes are mainly located in the hilus and ML but not in the GCL. These cells show brown DAB-staining in their processes and cytoplasm whereas the nucleus is devoid of staining (arrow). Calibration bar: 10 µm. H. Double immunofluorescent staining (orthogonal planes) of a DCX-Ki67 double immunopositive cell in the SGZ of a 21ds CORT+d18 MIF treated animal, demonstrating that at least a subset of the DCX^+^ cells can re-engage in cell cycle. Arrow indicates red Ki-67 signal in the nucleus of a green DCX^+^ cell. 40× magnification; GCL: granule cell layer of the hippocampal dentate gyrus.

BrdU was injected on day 1 and evaluated on day 18 (experiment #1) or day 22 (experiment #1 and 2). This gives insight in the survival of proliferating cells [39]. BrdU^+^ cells prevailed in the subgranular zone (SGZ; see Figure 2A for typical example) but were also found in the hilus and -in considerably lower numbers- the granule cell layer (GCL).

Doublecortin (DCX) is a microtubule binding protein expressed in young neurons from approximately 4 to 14 ds after birth of the cell [40]. DCX^+^ somata were located mainly in the SGZ and their processes extended through GCL into the molecular layer (shown in Figure 2B). In general, ‘gaps’ in the continuous line of DCX expressing cells in SGZ and shorter extensions were frequently found in CORT-treated animals compared to controls. We distinguished morphologically different subtypes of DCX^+^ neurons, reflecting different stages of neuronal development, as described before [37]: those without dendrites or with horizontally orientated dendrites were designated as type 1 cells (shown in Figure 2C) and cells with dendrites growing into GCL but not the molecular layer as type 2 cells (Figure 2D); these together are considered to be less mature neurons which can still undergo cell division [41]. In contrast, more mature DCX^+^ neurons are characterized by a primary dendrite orientated perpendicularly to the SGZ and with protrusions into the molecular layer (type 3 cells, Figure 2E).

Proliferating cell nuclear antigen (PCNA) is involved in leading strand synthesis during DNA replication and as such commonly used as a marker for cell division [42], [43]. In accordance with the literature [44], PCNA^+^ cells prevailed in SGZ but were also found in the hilus and to a lesser extent in the outer GCL as clusters, with multiple cells per cluster (typical examples in Figure 2F).

We further included quantification of the number of GFAP-positive astrocytes, as these glia cells have been implicated in the pathogenesis of affective disorders [45] and were found to be reduced after stress in animal models [46] and in patients suffering from depression [47], [48], [49]. Typically, GFAP^+^ cells showed brown DAB-staining in the processes and cytoplasm while the nucleus was devoid of staining and they were present throughout the main hippocampal subregions (Figure 2G).

### Effects of 17 versus 21 days treatment with corticosterone

Body weight gain was significantly different among the four experimental groups (ANOVA F(3,20) = 113.8, p<0.001; Table 1). Post-hoc analysis revealed that both 17 days and 21 days CORT exposure significantly (p<0.001) reduced the percentual change in body weight, compared to the respective vehicle controls. As expected, the gain in body weight was significantly lower after 17 ds VEHC than after 21 ds VEHC (p<0.05).

**Table 1:** Percentual change in body weight.

Group names	VEHC	CORT
Experiment 1		
21 ds+ds 18–21 VEHM	36.0±2.0	−4.5±3.0^a^
17 ds	27.5±0.9^b^	−0.1±0.6^a^
Experiment 2		
21 ds+ds 18–21 VEHM	36.0±2.0	−4.5±3.0^a^
21ds+d18 MIF	31.4±0.9	4.2±0.8^a^^,^ d
21ds+ds 18–21 MIF	43.1±1.0^c^	−0.6±2.1^a^

Corticosterone (CORT) compared to vehicle treatment (VEHC) reduced the percentual change in body weight, at 17 and 21 ds. This was not consistently affected by treatment with mifepristone (MIF).

a : significantly different from the corresponding VEHC group (p<0.001).

b : significantly different from the 21 ds VEHC+ds 18–21 VEHM group (p<0.05).

c : significantly different from the 21 ds VEHC+d18 MIF group (p<0.001).

d : significantly different from the 21 ds CORT group (p<0.05).

**Figure 3:**
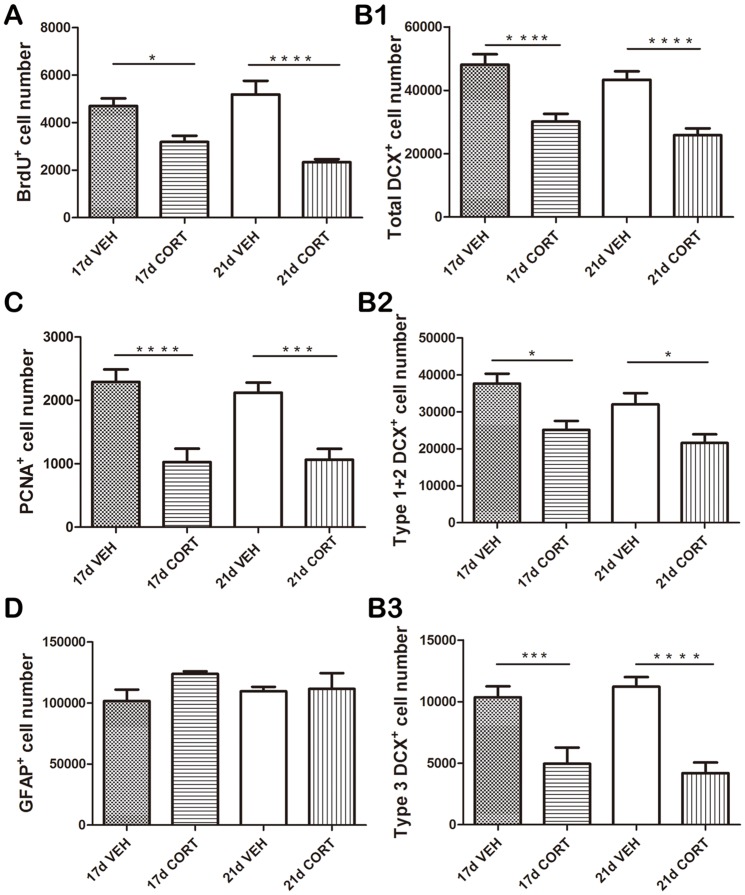
Effects of 17 versus 21 days treatment with corticosterone. **A.** Both 17 ds (p<0.05) and 21 ds CORT (p<0.001) exposure significantly reduced the number of BrdU^+^ cells compared to the respective vehicle control groups. **B1.** Likewise, both 17 ds and 21 ds CORT exposure significantly (p<0.001) reduced the total number of DCX^+^ cells compared to the control groups. **B2.** A significant reduction in immature DCX^+^ cells was found after 17 ds CORT (p<0.05) as well as after 21 ds CORT exposure (p<0.05) compared to the respective control groups. **B3.** The number of mature DCX^+^ cells was significantly reduced after 17 ds CORT (p<0.005) and after 21 ds CORT exposure (p<0.001) compared to the respective control groups. **C.** Both 17 ds and 21 ds CORT exposure groups showed a significant (p<0.001 and p<0.005 respectively) reduction in the number of PCNA^+^ cells compared to the corresponding control groups. **D.** Treatment with corticosterone did not affect the number of GFAP^+^ astrocytes at all (p>0.05). Data are presented as mean+SEM (n = 6 animals per group). For each marker, the groups were first subjected to an ANOVA, followed by a post-hoc Tukey multiple comparison of the means. * p<0.05; ***p<0.005; **** p<0.001.

The four experimental groups differed significantly from each other (F(3,20) = 13.7, p<0.001) with respect to the number of BrdU^+^ cells (Figure 3A). Post-hoc analysis showed that both 17 ds (p<0.05) and 21 ds CORT (p<0.001) exposure significantly reduced the number of BrdU^+^ cells compared to the respective controls.

Treatment also significantly affected the total number of DCX^+^ neurons (F(3,20) = 15.5, p<0.001; Figure 3B1). Both 17 ds CORT exposure (p<0.001) and 21 ds CORT exposure (p<0.001) significantly reduced total number of DCX^+^ neurons compared to controls at the same day. Treatment effects were reflected among the immature (type 1+2; ANOVA: F(3,20) = 8.2, p<0.001; Figure 3B2) as well as more mature (type 3; F(3,20) = 13.6, p<0.001; Figure 3B3) DCX^+^ cells. For both immature and mature DCX^+^ cells, the reduction in cell number was significant after 17 ds CORT (p<0.05 and p<0.005 respectively) and after 21 ds CORT exposure (immature neurons and mature neurons: p<0.05 and p<0.001) compared to the respective control groups.

The number of PCNA^+^ was also significantly (F(3,20) = 13.0, p<0.001) affected by treatment (Figure 3C). The 17 ds and 21 ds CORT groups were highly comparable and both showed a significant (p<0.001 and p<0.005 respectively) reduction in the number of PCNA^+^ cells compared to the corresponding control groups. As shown in Figure 3D, CORT treatment did not affect the number of GFAP^+^ astrocytes at all (F(3,20) = 0.977, p>0.05).

For all parameters tested (BrdU, DCX, PCNA, GFAP), the two VEHC groups (17ds VEHC and 21ds VEHC+d18–21 VEHM) were highly comparable. Similarly, the 17ds CORT and 21ds CORT (+d18–21 VEHM) groups did not differ significantly from each other for any of the markers investigated.

Collectively, these results are not compatible with the idea that reduction in adult-born cell number starts only >17 ds after the onset of CORT exposure, so that mifepristone at that time would exert a ‘rescue’ effect. Rather, our data indicate that throughout its presence, corticosterone steadily attenuates adult neurogenesis (see further Discussion).

### Effects of mifepristone administered on d18 or ds18–21 of a 3-weeks corticosterone treatment period

We next examined if we could replicate the earlier findings with mifepristone [36]; and if so, whether the chosen 4-days treatment period is required for complete reversal, or whether a single-day treatment at d18 can already ‘reset’ the corticosterone-induced attenuation of proliferation. To this end, we compared six experimental groups, which differed from each other with respect to corticosterone treatment (CORT vs VEHC for 21 ds) and mifepristone treatment (a single administration of mifepristone on d18; mifepristone on 4 consecutive days, i.e. ds 18–21; no mifepristone, i.e. VEHM on ds 18–21). An overall ANOVA revealed a significant effect on the percent change of body weight (F(5,30) = 132.7, p<0.001; Table 1), which was mostly explained by a significantly reduced body weight in all 21 ds CORT groups, with or without mifepristone (p<0.001 in all cases). In the VEHC groups, 4 days of mifepristone administration resulted in more gain in body weight than in the group which received no mifepristone at all. Moreover, the 21ds CORT+d18 MIF group had less attenuated body weight gain than the CORT treated group receiving no mifepristone.

**Figure 4:**
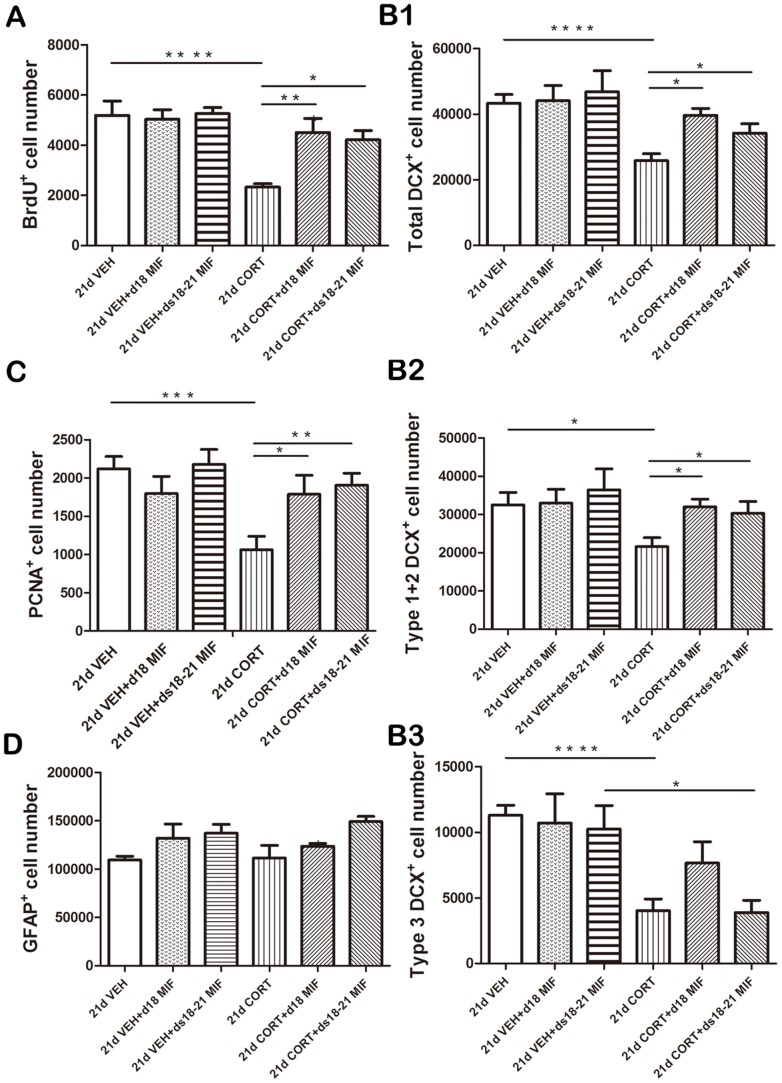
Effects of mifepristone given at d18 only or during ds18–21 of 3-weeks corticosterone treatment period. **A.** Mifepristone treatment during ds 18–21 fully normalizes the CORT-induced reduction in number of BrdU^+^ cells (p<0.05 compared to 21 ds CORT+d18–21 VEHM). Surprisingly, mifepristone treatment only on d18 also normalized the CORT-induced reduction (p<0.01). No significant difference was found between these two treatment groups (21ds CORT+ds18–21 MIF versus 21ds CORT+d18 MIF; p>0.05). Mifepristone treatment was entirely ineffective in the vehicle (VEHC) control groups. **B1.** Mifepristone treatment either during ds 18–21 (p<0.05) or only on d18 (p<0.05) normalized the CORT-induced reduction in DCX^+^ cells. These two treatment groups (21ds CORT+ds18–21 MIF versus 21ds CORT+d18 MIF) yielded comparable numbers of DCX^+^ cells. **B2.** Mifepristone treatment either during ds 18–21 (p<0.05) or only on d18 (p<0.05) was effective in normalizing the CORT-induced reduction in immature DCX^+^ cells. **B3.** Mifepristone was ineffective in restoring the CORT-induced reduction in the number of mature DCX^+^ cells (p>0.05 in both treatment groups). CORT-treated animals receiving mifepristone for 4 days (21ds CORT+ds18–21 MIF) still had a significantly lower number of mature DCX^+^ cells than the corresponding vehicle group (p<0.05 vs 21ds VEHC+ds18–21 MIF). **C.** Mifepristone administration either on d18 alone (p<0.05) or during ds18–21 (p<0.01) normalized CORT-induced reduction in PCNA^+^ cells. Again, mifepristone treatment had no effect in the VEHC groups. **D.** No significant overall effect of treatment was found on GFAP^+^ cells number (p>0.05). Data are presented as mean ± SEM (n = 6 animals per group). For each marker, the groups were first subjected to an ANOVA, followed by a post-hoc Tukey multiple comparison of the means. * p<0.05; ** p<0.01; ***p<0.005; **** p<0.001.

The number of BrdU^+^ cells differed among the groups (F(5,30) = 7.3, p<0.001). We completely reproduced our earlier finding [36] that mifepristone treatment during ds 18–21 fully normalizes the CORT-induced reduction in number of BrdU^+^ cells (p<0.05 compared to 21 ds CORT+d18–21 VEHM; Figure 4A). Surprisingly, mifepristone treatment only on d18 also normalized the CORT-induced reduction (p<0.01) and no significant difference was found between these two treatment groups (21ds CORT+ds18–21 MIF versus 21ds CORT+d18 MIF; p>0.1). Mifepristone treatment was entirely ineffective in the vehicle (VEHC) control groups.

A highly similar pattern was observed with respect to the number of DCX^+^ cells: We observed a significant overall difference between the groups in the number of DCX^+^ cells (F(5,30) = 4.2, p<0.01). Mifepristone treatment either during ds 18–21 (p<0.05) or only on d18 (p<0.05) normalized the CORT- induced reduction in DCX^+^ cells (Figure 4B1). These two treatment groups (21ds CORT+ds18–21 MIF versus 21ds CORT+d18 MIF) yielded comparable numbers of DCX^+^ cells. Follow-up analysis of various developmental stages in DCX^+^ cells showed that mifepristone treatment was effective in normalizing the CORT-induced reduction of immature DCX^+^ neurons to control levels (ANOVA: F(5,30) = 4.4, p<0.005; Figure 4B2). Both the 21ds CORT+d18 MIF and 21 ds CORT+ds18–21 MIF groups differed significantly (p<0.05) from the 21ds CORT+ds18–21 VEHM group. The number of mature DCX^+^ cells was also different among the treatment groups (F(5,30) = 5.2, p<0.005; Figure 4B3). However, post-hoc analysis of the groups showed that mifepristone was ineffective in restoring the CORT-induced reduction in the number of mature DCX^+^ cells. CORT-treated animals receiving mifepristone for 4 days (21ds CORT+ds18–21 MIF) still had a significantly lower number of mature DCX^+^ cells than the corresponding vehicle group (p<0.05 vs 21ds VEHC+ds18–21 MIF). This group did not differ significantly (p>0.05) from the CORT-treated group receiving MIF only on d18.

A similar overall pattern was observed for the proliferation marker PCNA (ANOVA: F(5,30) = 6.6, p<0.001; Figure 4C). Thus, mifepristone administration on d18 alone (p<0.05) or during ds18–21 (p<0.01) normalized CORT- induced reduction in PCNA^+^ cells; in this respect the two mifepristone-treated groups were indistinguishable. Again, mifepristone treatment had no effect in the VEHC groups. We did not observe a significant overall effect of treatment on GFAP staining (ANOVA: F(5,19) = 2.649, p>0.05; Figure 4D).

Double immunofluorescent staining for DCX and the proliferation marker Ki-67 revealed several co-labeled cells in the 21ds CORT+d18 MIF group indicating that a subset of the neurogenic cells is actively engaged in proliferation after MIF treatment (see example in Fig. 2H). This was not observed in the vehicle-treated control group (not shown).

In conclusion, for all proliferation and survival markers, mifepristone treatment on d18 alone was as effective as treatment on ds18–21.

## Discussion

This study set out to elaborate on our previous findings that a GR-antagonist can rapidly reverse the reductions in adult neurogenesis caused by chronic stress exposure [35] or prolonged corticosterone administration [36]. In the current study, we replicated this phenomenon and provide evidence that GR-antagonist mifepristone does not achieve its effect by rescuing vulnerable cells from a late cell death, but rather by reversing the ongoing corticosterone-induced attenuation of cell proliferation, and thereby stimulating extra rounds of cell division. Surprisingly, also a single day treatment on day 18 had exactly the same effect as when the drug was given for 4 consecutive days.

### Experimental design

We chose to treat rats with corticosterone for 21 days, rather than exposing them to daily stress. While daily corticosterone treatment is essentially different from chronic stress, the common element in both paradigms is the extended over-exposure to corticosterone. Since both approaches cause strongly overlapping effects on neurogenic process [35], [36], corticosterone seems to be an important mediator. We therefore selected the more straightforward (and easier to accomplish) protocol of corticosterone administration for 21 days. Extrapolation of these findings to conditions of chronic stress, however, should be done with care.

The phenotype of 21ds corticosterone administration is robust. Earlier experiments had shown that this regime strongly reduces body weight gain as well as adrenal and thymus weight [36], [50]. We here only examined body weight gain and observed a severe attenuation in body weight gain in all corticosterone treatment groups, i.e. also after 17ds of hormone administration. We did observe some effects of mifepristone on body weight, but these were only small and not entirely consistent. For instance, in corticosterone-treated animals mifepristone administration on d18 caused a less severe attenuation in body weight gain compared to animals receiving no mifepristone, but this was not seen with mifepristone treatment for 4 consecutive days.

Mifepristone has a strong affinity for the GR, preventing transactivation of GR-responsive genes [51]. However, this compound also binds to progesterone receptors. Although the anti-progesterone activity is likely limited in adult male rats, we cannot entirely exclude some of the observed affects were due to mixed pharmacological profile of mifepristone. We nevertheless decided to use this drug, to allow easy comparison with earlier studies in rats and men [11], [12], [13], [14], [35], [36], [51], [52], [53], [54], [55], [56], [57].

A potential limitation of the present study is that an extra experimental group treated with vehicle only on d18 was not included. This was done as a 21ds CORT+ds18 VEHM group was expected not to be different from the 21ds CORT+ds18–21 VEHM animals, which we did examine. Although one could reason that 4 days of (oral) VEHM administration is more stressful than just 1 day, it should be noted that all animals already received an injection (CORT or VEHC) in the morning, so that the added stress of VEHM delivery was probably limited. Moreover, in the evening (during the 2^nd^ MIF administration) CORT levels are at the peak of circadian rhythm, so that even in the VEHC group the relative increase in corticosterone level due to VEHM delivery was probably low. Therefore, we expect administration of VEHM by itself (be it once or on 4 consecutive days) will not have influenced our data much. This is in line with the fact that both a single- and 4 consecutive-days administrations of mifepristone in the VEHC groups did not affect any of the parameters investigated; if the administration of mifepristone (or its vehicle) was highly stressful, we would expect that a single versus repetitive exposure causes a different phenotype, which was not the case. Altogether we have no reason to assume that lack of the VEHC/CORT+d18 VEHM groups would seriously hamper our conclusions.

### Primary target of corticosterone in the neurogenic pathway

Earlier studies have supplied evidence that chronic corticosteroids over-exposure affects multiple steps in the neurogenic pathway: corticosteroids reduce proliferation [29], decrease neuronal differentiation [31], and impair survival of adult-born cells [32], [36].

Our first experiment gives additional insight in this issue. The results with PCNA -a marker for cell proliferation at the time of sacrifice [58], [59]- indicate corticosterone exposure reduces proliferation, both after 17 ds and 21 ds treatment. The degree of reduction was comparable at both timepoints, suggesting an ongoing attenuation during the daily corticosterone exposure. Immunostaining for DCX reflects the sum of neuronal differentiation and survival of migratory young neurons born 4–14 days before staining [40]. Given the attenuated proliferation indicated by PCNA staining on d18, the reduction in number of immature DCX^+^ cells seen on d22 (partly reflecting cells that were born around d18) could be well explained by a reduced proliferation of cells that subsequently differentiate into neurons. In view of the highly comparable reduction in the number of immature DCX^+^ cells at d18 (which were most likely born <d14), corticosterone treatment may indeed suppress proliferation for most of the 21ds treatment period. This is underlined by the extensive reduction in number of mature DCX^+^ cells (a cumulative measure of cells born during the first two weeks of treatment and differentiating into neurons), both after 17 and 21 ds corticosterone exposure. Nevertheless, additional corticosterone effects on neuronal differentiation and/or survival cannot be excluded. A shift from neuronal into glia phenotype seems unlikely, because the number of GFAP^+^ cells is not altered; however, it is possible that changes in differentiation may remain unnoticed, since the number of newborn cells becoming GFAP^+^ is only a fraction of the total pool of GFAP^+^ cells [60], [61], [62].

In our experimental design, BrdU staining reflected the survival of cells that were derived-probably after several rounds of replication- from a subgroup of cells born on day 1. If we assume an ongoing suppression of replication by corticosterone, the reduced number of BrdU^+^ cells both at d18 and d22 can be understood in the absence of any steroid effect on cell survival or even with increased cell survival. However, if cells incorporating BrdU on d1 did not undergo several rounds of replication, the findings support the view that corticosterone impairs survival of newborn cells.

Collectively, although additional hormonal influences on differentiation and survival of newborn cells cannot be excluded, the results support the view that corticosterone attenuates cell proliferation and adult neurogenesis, probably for most of period in which the hormone is administered.

### Effects of mifepristone

The findings with PCNA indicate that mifepristone normalized corticosterone-induced attenuation in cell proliferation. As the antagonist was ineffective in VEHC-treated animals, we conclude that mifepristone -rather than e.g. through additional pathways- prevents corticosterone from exerting suppressive effect on proliferation. Surprisingly, this was not only seen when mifepristone was administered during the days preceding the moment on which proliferative activity was probed (d22), but even when it was given just on d18, i.e. 4 days before PCNA staining. Thus, despite continued presence of corticosterone after d18 and the presumed absence of mifepristone at that time- its presence at d19–21 can be ruled out in view of the short half-life time [63], [64]- later corticosterone no longer seems able to reduce proliferation or overrule the proliferative changes initiated at d18.

The observations with DCX are also compatible with the view that mifepristone prevents corticosterone not only from reducing proliferation, but also from reducing the number of cells that lateron develop into neuronal phenotype. The number of immature DCX^+^ cells (which were born on d18 or earlier) was fully restored by mifepristone. The fact that corticosterone-induced reduction in the number of mature DCX^+^ cells was not normalized fits with this view, since the proliferation giving rise to these cells probably took place before mifepristone was administered. Incidentally, if mifepristone would primarily prevent a putative corticosterone-induced suppression in cell survival, one would expect that the number of mature DCX^+^ cells is also (partly) normalized by mifepristone; since the number of mature DCX^+^ cells in the 21ds CORT+ds18–21 MIF group was significantly lower than that in the 21ds VEHC+ds18–21 MIF group, this strongly argues against effects of corticosterone and mifepristone on cell survival per se. Similar to the PCNA results, mifepristone on d18 alone was as effective as a 4ds delivery in normalizing the number of immature DCX^+^ cells. This is less surprising, since DCX immunoreactivity discerned on d22 most likely reflects neurons born at d18 or earlier.

A significant proportion of the newborn cells in adult SGZ undergoes apoptosis, most likely within their first week of life [39], [65]. Earlier studies have suggested that GR activation may play a pivotal role in glucocorticoid-induced apoptosis and proliferation [66], [67], whereas mifepristone pre-treatment could e.g. prevent stress-induced apoptosis of hippocampal newborn neurons [56]. Albeit in osteoblastic cells, mifepristone also abolishes the GR agonist dexamethasone (DEX)-induced apoptosis and G0/G1 arrest and increases cell proliferation, an effect that may be mediated through GR [68]. At the start, we considered the possibility that mifepristone may exclusively act by preventing death of a group of neurons [56] at d18 or later. If so, we would expect the number of BrdU^+^ cells to be comparable up to d18 and then drop dramatically. However, this was not the case (experiment #1). Since the equilibrium between proliferation and death of cells born on d1 (reflected by the number of BrdU^+^ cells) can be fully restored by mifepristone, and in view of the findings with PCNA and DCX, we conclude that normalization in the number of BrdU^+^ cells at d22 by mifepristone is caused by the drug preventing attenuation of proliferation caused by corticosterone. In fact, mifepristone may, either directly or indirectly, also stimulate proliferation of some neurogenic cells. As shown in Fig. 2H, a small subset of the DCX^+^ cells were co-labeled for the proliferation marker Ki-67 indicating that at least some of the neurogenic cells can re-engage in proliferation. Earlier, Walker et al. [69], using fluorescence-activated cell sorting (FACS), could demonstrate that of the DCX^+^ cells those with relatively low levels of DCX per cell were capable of dividing again.

As was argued above for PCNA, the BrdU data support the concept that a single mifepristone administration is sufficient to prevent subsequent corticosteroid-effects at least for several days, even in the continued presence of corticosterone. Regarding this rapid normalization, several similar observations exist in literature in which e.g. the HPA axis or opioid system were ‘reset’ already after a short stimulus; for example, a short inescapable stressor produced long-lasting changes in the brain-pituitary-adrenal axis of adult male rats, while a single administration of interleukin-1 causes long-lasting changes in HPA sensitization [70], [71], [72]. Moreover, also for opioid sensitivity, similar ‘swicth-like’ effects have been described [73], [74], [75].

In conclusion, the current study provides evidence that repetitive corticosterone administration primarily attenuates proliferation and more specifically neurogenesis, in the rat DG. This is prevented already by a brief administration of a GR-antagonist. Surprisingly, also a single day administration of the antagonist is already sufficient to normalize the neurogenic process, possibly by resetting their initial level of proliferation. A similar quick switch may explain the rather rapid beneficial effects of mifepristone observed in a small sample of patients with psychotic depression [12], [14], [54], [55]. This underlines that studies into therapeutical efficacy of experimental antidepressants that target HPA-activity should explore possibility of reducing treatment duration since this might result in equally beneficial effects.
